# Development of a Scaffold from the Cob of *Zea mays* L. “Choclo” to Obtain an In Vitro Bone Tissue Model

**DOI:** 10.3390/jfb17060267

**Published:** 2026-06-01

**Authors:** Rafael Carbajal-Valverde, Luz Pérez-Tulich, Franco Huaccha-Cáceres, Giulianna Travi-Antonio, Julio Valdivia-Silva

**Affiliations:** Centro de Investigación en Bioingeniería, Universidad de Ingenieria y Tecnologia (UTEC), Lima 15063, Peru

**Keywords:** bone tissue engineering, decellularized scaffold, *Zea mays* L., bone model, functionalized plant tissue

## Abstract

The lack of accessible in vitro 3D bone tissue models puts developing countries at a disadvantage in terms of research capacity and healthcare access. In this study, a decellularized corn cob scaffold was functionalized with GTMAC by adding quaternary ammonium groups and coating it with an alginate–gelatin hydrogel to promote mesenchymal stem cell adhesion, offering a low-cost platform for bone disease modeling. Systematic characterization of the scaffold demonstrated that decellularization reduced DNA content by over 80%. Chemical treatment affected the mechanical properties of the matrix, while the hydrogel coating and the functionalized surface of the scaffold promoted cell adhesion and morphology comparable to that observed in 3D cell cultures. MTT analysis showed a subtle reduction in metabolic signal in functionalized scaffolds, which may reflect changes in cell distribution and adhesion within the 3D matrix rather than cytotoxic effects.

## 1. Introduction

Novel biomaterials for 3D in vitro culture aim to replicate the native cellular microenvironment in which cells grow [1,2]. Bone tissue engineering seeks to regenerate tissue-mimetic materials to promote bone regeneration in treatments; the scaffolds created must have similar mechanical properties and biocompatibility to what is sought in the tissue [3,4]. Biomimetic 3D constructs have applications in in vitro drug testing and disease research, because cells behave differently when being cultured in 2D than in 3D; for example, osteoblasts are used in a 3D environment, such as bone tissue. 3D culturing is a physiologically relevant model because it triggers more accurate gene expression, as demonstrated in the study carried out by Payr S., where elderly osteoblasts cultured in a polystyrene scaffold expressed a greater number of different markers—Osteoprotegerin, collagen-1, and Osteopontin—than a regular 2D culture [5]. Cells cultured on flat 2D surfaces, however, have the ability to re-acquire their three-dimensional shape when introduced into a 3D scaffolding environment [6].

The biomaterials used regularly, like collagen, are functional and readily available, but become expensive and lack the mechanical properties of bone when turned into hydrogels and not enhanced with other composites [7,8]. In addition, the use of animal-derived biomaterials has become an ethical concern due to the process used to obtain them, and can be very expensive because it requires specific equipment when developing 3D structures [9].

Key developments in scaffold fabrication techniques, including additive manufacturing and the incorporation of bioactive molecules, aim to mimic the natural microenvironment of bone, thereby enhancing osteogenesis and facilitating new tissue formation [10]. Within this context, plant-derived scaffolds have attracted growing interest as sustainable, cost-effective, and ethically favorable alternatives. Plant-derived scaffolds present inherent structural diversity, widespread availability, and a lower risk of zoonotic disease transmission; in particular, cellulose—a major structural component of plant cell walls—offers excellent mechanical strength, porosity, and hydrophilicity that make it suitable for biomedical applications [11]. This field has expanded beyond its initial tissue engineering scope to include tissue models, drug testing platforms, and biosensors, with a threefold increase in related publications between 2020 and 2024, reflecting accelerating research interest [12]. In the context of bone repair specifically, decellularized plant cellulose scaffolds have been shown to support the growth and osteoblastic differentiation of pluripotent stem cells, with resultant mineralized constructs successfully implanted in rat calvarial defect models where they promoted calcified tissue formation [13]. Similarly, decellularized cabbage scaffolds seeded with bone-marrow-derived mesenchymal stem cells have demonstrated significantly elevated ALP activity, mineralization, and gene expression of osteocalcin, collagen-1, RUNX2, and ALP compared to cells cultured on standard tissue culture plates [14]. Despite these advances, ongoing research continues to refine decellularization protocols and address mechanical limitations and the absence of vascular networks, which remain key barriers to clinical scalability and widespread adoption [15].

Scaffolds in which osteoblasts are seeded are an alternative as a study model to treat bone conditions, like pseudoarthrosis, bone loss, among others; they also have applications in salvage surgery for patients with bone tumors, bone replacement surgery, and bone grafts [16]. For this reason, plant tissues have recently been used for the production of scaffolds in which osteoblasts are cultured; attempts have also been made to generate bone models that imitate the nature of the bone tissue [9], for which apples, carrots, or bamboo have been investigated for this purpose through tissue decellularization [14]. This procedure helps to reduce manufacturing costs and has shown favorable results in in vitro tests and in animals, such as mice [15].

A promising opportunity has been identified in the development of a novel 3D plant-based bone tissue model derived from the cob of *Zea mays* L. (commonly known as corn). Peru produces approximately 78,000 tons of corn annually, yet in 2017 it was recorded that 45% of national production generated non-reused waste—including the cob itself [17]—positioning this material as both an abundant and sustainable local resource. Notably, the Peruvian “capia” (white maize) variant is among the most widely cultivated and consumed in the country, ensuring consistent material availability.

The *Zea mays* cob is composed of three distinct layers: the pith, the glume, and the woody ring. The pith and glume present a sponge-like porous architecture with pore sizes ranging from 100 to 160 µm, while the pith exhibits a Young’s modulus of 7.62 MPa and the whole cob a Young’s modulus of 59.4 MPa [18]—among the highest values reported for scaffolds of vegetal origin [18,19]. These structural and mechanical properties align with the requirements for osteoblast culture, which demands pore sizes between 50 and 200 µm and substrate stiffness values similar to bone. Ogurkowska reported that the Young’s modulus of trabecular bone from the vertebral body ranges from 26.39 MPa to 74.57 MPa, though the mean Young’s modulus of larger, less anisotropic bones ranges from 3.5 to 18 GPa [20,21,22,23,24,25,26,27]. Furthermore, the cob’s primary chemical constituent—cellulose—offers a versatile surface amenable to chemical functionalization, which can be leveraged to enhance cell adhesion and matrix–cell interactions [28].

Decellularization of the cob provides a cost-efficient strategy to preserve this native mechanical architecture while rendering it suitable as a 3D cell culture matrix, without the requirement for specialized or expensive equipment [29]. Taken together, these characteristics position the *Zea mays* cob as a strong candidate scaffold for osteoblastic cell culture, with potential application in complex in vitro 3D bone tissue research—particularly in studies examining the influence of three-dimensional structures and mechanical stimuli on osteoblast gene expression.

## 2. Materials and Methods

**Preparation of the Scaffold:** The scaffold produced was extracted from a corn cob that we acquired from a local market; the sample was sliced to dimensions of 3 cm in height × 2 cm in diameter, preserving the 3 layers: pith, glume, and woody ring [18]. After obtaining this slice through radial cutting, we subjected it to a process of freezing and thawing for 24 h, performing this process twice at −70 °C [29,30,31]. We submerged the piece into 5% SDS (CAS 151-21-3, purchased from Merck, Sigma-Aldrich Inc., St. Louis, MO, USA) for 24 h, and changed the solution once every 2 days for 5 days. Afterwards, the sample was washed thoroughly with dH_2_O for removal of the bleach for 2 days; the water was changed every 8 h [32]. Once the scaffold is prepared, it may be stored at −4 °C suspended in 70% ethanol, PBS, or dH_2_O until its use.

To use the scaffold, it should be prepared one day prior. In this study, it was sterilized in an autoclave at 121 °C for 15 min and submerged in 70% ethanol for 6 h, then a 1–2% antifungal antibiotic—streptomycin, penicillin, and amphotericin [100 μg/mL] (SIgma-Aldrich, St. Louis, MO, USA)—was applied at 37 °C, and it was placed at 200 rpm in a shaker [14,15].

Other decellularization methods were also performed on the cob, using varying surfactants and reactants; Triton X (EMD millipore corp., Burlington, MA, USA), NaOH, and H_2_O_2_ were the other chosen solutions [29,30,31].

**Water Content of the Scaffold (WC):** In order to acquire the weight of the dry decellularized scaffold, the following equation may be used to calculate the relative humidity of the treated cob:

$$\%WC=\frac{Tw\;-\;Dw}{Dw}\times 100$$

where *Tw* is the total weight of the scaffold when dried softly with paper tissue, and *Dw* is the dry weight of the scaffold after being exposed to 60 °C for 24 h.

**DNA Concentration after the Decellularization Process:** After decellularization of the samples, a GeneJET Plant DNA Purification Kit (Thermofisher Scientific, Vilnius, Lithuania) was used to determine the amount of DNA remaining after the decellularization process, from cut samples of 2 cm^2^ that were pulverized with dry nitrogen and had a dry weight of 0.27 ± 0.2 g [33]. The DNA was eluted in 100 μL of elution buffer.

**Scaffold Functionalization and Coating:** After the decellularization process, the scaffold was placed inside a zip bag with 5% wt NaOH of the cellulose weight of the cob [34]. Since the cob is approximately 40% cellulose, the total weight must be taken from the dry weight of the scaffold [35]. The amount of solution must humidify the whole cob by covering it; then, 0.6 g of glycidyltrimethylammonium chloride (GTMAC) (SIGMA-ALDRICH., St Louis, MO, USA) was added, and it was placed in a water bath for 3 h at 65 °C, with occasional hand kneading [34,36]. To stop the reaction, 90° ethanol was poured directly on the scaffold, and it was placed in the shaker at 200 rpm at 37 °C, changing the medium every hour for 6 h; the solution was then discarded [34,36]. The scaffold was washed with dH_2_O at least 6 times or until the medium appeared clear and the pH reached physiological levels (pH: 7.4); PBS 1Xmay be used instead of distilled water [34]. The scaffold was coated with a 4–6% alginate–gelatin hydrogel (crosslinked with Cl_2_Ca 0.1 M, for 1 h) to improve cell adherence, with coating adhesion to the scaffold surface enhanced through electrostatic interactions [37]. The resulting construct is referred to as “Construct” throughout this paper.

**Cell Expansion and Storage:** Cryopreserved human mesenchymal stem cells (hMSCs), obtained from an umbilical cord from a donor in 2019, were cultured in DMEM (Life Technologies Corporation, New York, NY, USA) supplemented with 10% FBS in T25, T50 at 37 °C and 5% CO_2_. A million cells were used for cell expansion in T125 plates at 37 °C, 5% CO_2_ until 80% confluence; the used media were DMEM containing 10% FBS and 1% streptomycin–penicillin. The leftover cells that were not used for experiments were stored in 10% DMSO (SIGMA-ALDRICH., St Louis, MO, USA) at −70 °C [38,39].

**Chemical and Structural Characterization of the Scaffold:** Both decellularized and functionalized scaffolds, as well as a control, were dried, cut into tiny pieces, and then pulverized using a mortar and pestle with the help of liquid nitrogen to perform an FTIR read (Shimadzu, Nakagyo-ku, Kyoto, Japan) [34]. To study the tissue structure of corn cob, histology slides were prepared through fixation, dehydration, clearing, embedding, and sectioning; these processes were performed using a standardized laboratory protocol. Macrostructural images from the three layers of the tissue, stained with 1% Methylene Blue and Safranine, were obtained from Eurotech Digital Binocular Stereo Microscope—NSZ-405D (Eurolab, Hamburg, Germany).

**Cell Culture and Differentiation in the Scaffold:** Cells were differentiated in 96-well plates coated with 1% agarose; assays were performed in 4 groups that initially had DMEM as media: the first group was a control with media and cells; the second group contained the SDS-treated scaffold, media, and MSCs; the third group was the SDS-GTMAC-modified scaffold and cells; the last group was the gelatin–alginate hydrogel-coated SDS-GTMAC scaffold with cells. The scaffolds in the wells were exposed to the conditions found in the “Cell Expansion and Storage” section for 3 days before cells were introduced to the scaffold by immersion; this was achieved by pipetting on the top of the sample and filling up the well. When 80% confluence was reached in each well containing MSCs, the media were changed to osteoblast-inducing media, which contained DMEM, FBS 10%, 100 nM dexamethasone, 200 µM ascorbic acid (Fermont, Monterrey, Mexico), and 10 mM of glycerol 2-phosphate (ChemCruz, Dallas, TX, USA) [14,15,40]. With the help of a Zeiss Axio Vert.A1 LED fluorescence inverted microscope (Zeiss, Jena, Germany), the morphology and quantity of cells were evaluated during the proliferation and differentiation periods; observation of the cells was performed after thoroughly washing the scaffold twice with PBS 1X. DAPI staining was applied to visualize MSCs inside the scaffold [15]. The Alizarin Red (Sigma Aldrich, St Louis, MO, USA) staining procedure was performed using a 2% concentration; the scaffolds were observed under fluorescent light at an emission wavelength of 530 nm.

**Viability of Cells Exposed to the Scaffold:** To evaluate the cells’ response to the developed scaffold, an MTT assay was performed in ELISA plates (Elisa Bio-Rad Imark, Hercules, CA, USA). The experiment used 5 mm square samples obtained from the decellularized pith of the corn cob. Two scaffold conditions were evaluated: the SDS-decellularized pith and the SDS-decellularized pith followed by functionalization with GTMAC. Four groups were analyzed: a positive control (cells cultured without the scaffold), a negative control (PBS), and cells seeded onto the SDS-decellularized–autoclaved pith (Group A) and the SDS-functionalized–autoclaved pith (Group B). A total of 10,000 cells were seeded in a 96-well plate and allowed to adhere for 6 h, after which the scaffolds were placed in direct contact with the cell monolayer for 24 h. After using forceps to remove the scaffold from the wells with cells, 10 µL of the MTT reagent was added to each sample with cells and left to react for 4 h at 37 °C and 5% CO_2_. Then, 100 µL of the DMSO detergent was added and left to incubate at room temperature, protected from light, for 2 h. Finally, the samples were analyzed in the ELISA plate reader at 560 nm. Statistical analysis was performed using a Student’s *t*-test, with three repetitions and a significance level of 0.05 [41].

**Mechanical Testing:** Mechanical tests were performed on the cylindrical cob samples (1 cm height × 3 cm diameter) from an unthreaded cob (control), autoclaved control, and SDS-decellularized cob; all containing both the pith and woody ring with a relative humidity of 232.93%. Samples were prepared using a milling machine (Neway VM702S milling machine, Neway Valve, Suzhou, China) and a cutter to obtain multiple cylinders measuring 30 mm in length and 20 mm in diameter. Sandpapers with grit sizes of 240 and 280 were used to achieve a uniform cylindrical geometry and smooth surface finish. The tests were performed using the multipurpose mechanical test machine EXCEED Model E42 (MTS system, Västra Fröunda, Sweden) with the Pasco materials testing system base ME 8229. A radial quasi-static compression test was performed at a velocity of 0.2 mm/min; the test was run three times, and a normal curve was drawn [29]. ASTM D695 serves as a guide to calculate the apparent Young’s modulus (E) at the Hookean region of the curve using the equation E = *σ*/*ε*, where stress is represented as *σ* = *Applied force*/*Area of the sample* where the force is applied, and strain is represented as *ε* = *deformation of the axial height*/*original axial height* [42].

## 3. Results

**Figure 1:**
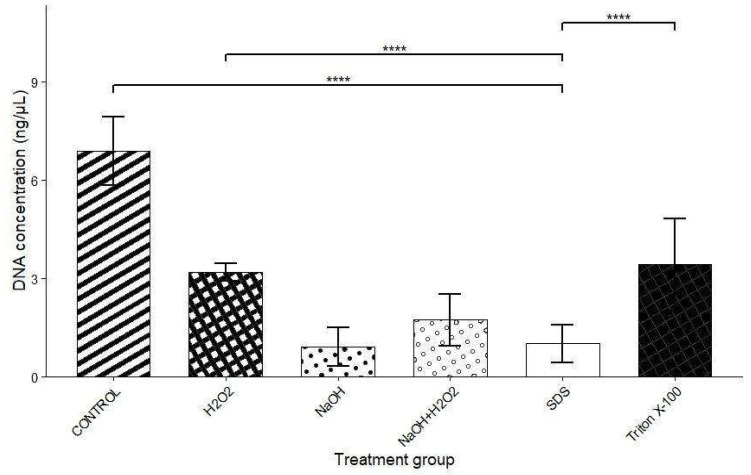
DNA quantity (ng/µL) of the cob of *Zea mays* L. after using different chemical decellularization methods (**** *p* < 0.05).

**Final DNA (ng/mL) After Decellularization and Modification of the Corn Cob Provides an Additive Cellulose Scaffold for the 3D Cell Culture:** All surfactants evaluated were able to decellularize the tissue to different extents. The percentage of DNA removal achieved by each treatment was as follows: Triton X-100 (50%), H_2_O_2_ (60%), NaOH and NaOH + H_2_O_2_ (70%), and SDS (80%). Among these treatments, SDS (0.26 ng dsDNA/mg) and NaOH (0.30 ng dsDNA/mg) showed the highest decellularization efficiency, suggesting that they are the most suitable reagents for preparing corn cob scaffolds as potential hosts for mesenchymal stem cells (MSCs). However, SDS was selected as the preferred treatment, as discussed in the Discussion Section. Figure 1 illustrates the efficiency of each decellularization method applied to the corn cob, showing that both SDS and NaOH effectively decellularize this hard–soft tissue within a short period using an immersion-based decellularization approach.

**Figure 2:**
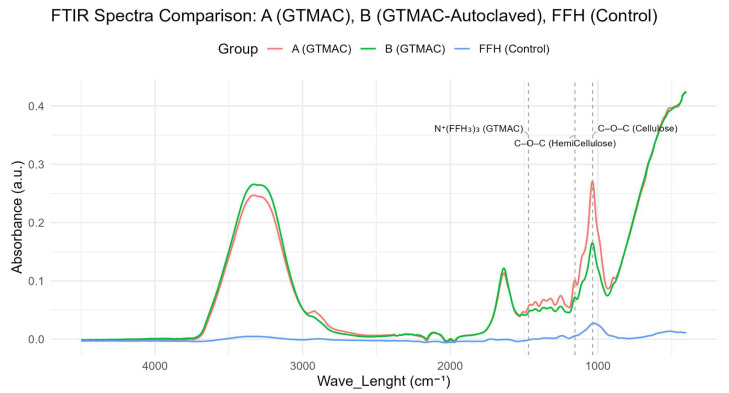
Zoomed FTIR of the differences between the absorbances of the GTMAC, GTMAC-autoclaved, and control groups.

**Figure 3:**
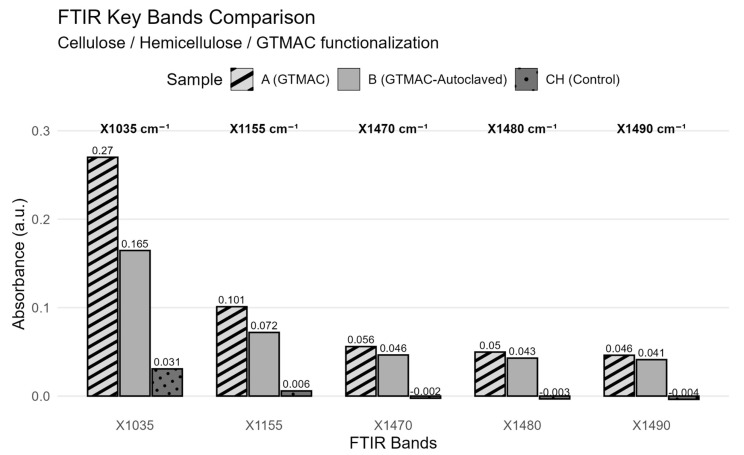
FTIR key bands comparison of the absorbance of the GTMAC, GTMAC-autoclaved, and control groups.

The alkaline modification of the scaffold was performed through the use of GTMAC. For this purpose, the relative humidity of the SDS-treated scaffold was 232.93%, in order to improve cell and hydrogel adhesion to the matrix. FTIR revealed small modifications in the regions at 1035, 1155, 1470–1490, and 1510–1520; the peaks are visualized in Figure 2, where 1470–1490 represents the addition of ammonium quaternary groups [34]. The zoomed analysis that was performed, and the differences in absorbance, are shown in Figure 3.

**Figure 4:**
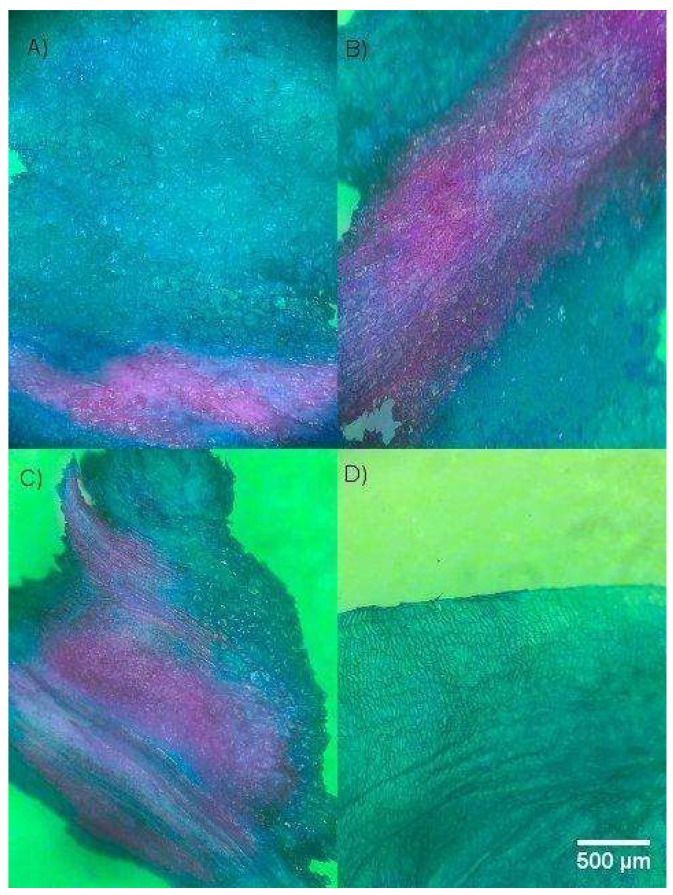
Safranin–Methylene Blue staining of the three sections of the corn cob under stereoscope: pith coronal section (**A**); xylem/phloem of the pith (**B**); woody ring (**C**); and glume (**D**).

**Structural Characterization:** The pith, woody ring, and glume of the decellularized–autoclaved cob are shown in Figure 4: the pith (Figure 4A) shows a non-lignified vegetal parenchymal structure that is not arranged or organized; the xylem/phloem in the cob can be seen as organized and oriented with a lignified parenchymal structure (Figure 4B); the woody ring has an arranged group of lignified parenchyma (Figure 4C); and the glume (Figure 4D) presents an organized cellulose structure. The purple-like color in all images represents the lignified parenchymal tissue that is present in the corn cob.

**Figure 5:**
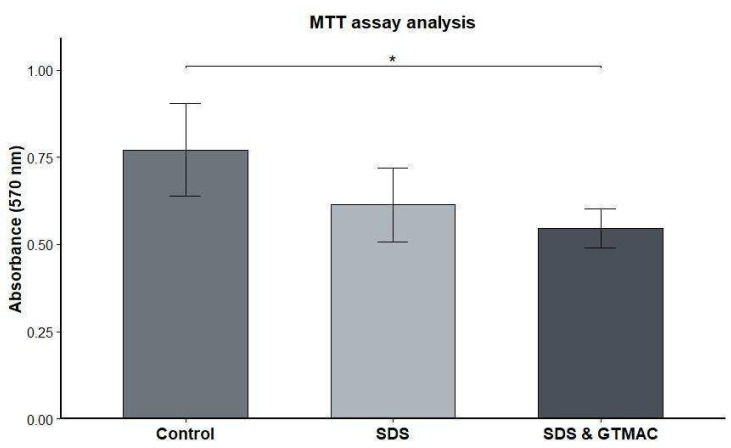
MTT analysis showing the absorbance of control and exposed hMSCs to the SDS and SDS-GTMAC scaffold groups (* *p* < 0.05).

**Cell Viability:** Cytotoxicity of the scaffold was measured by exposing hMSCs and performing MTT assays. It can be seen in Figure 5 that the SDS treatment was not cytotoxic to cells after 24 h of exposure, since there is no significant difference from the control (*p* < 0.05); however, SDS-GTMAC reduces cell viability (*p* > 0.05), as covered in the Discussion Section, where the reasons are explored.

**Relative Elastic Modulus of the Cob:** The corn cob went through mechanical testing, with compression forces applied along its axial axis. Three groups were evaluated: control, SDS, and autoclaved control; Figure S1 shows the initial part of the stress vs. strain graph. The three graphs display similar behavior across the groups, and their elastic moduli are shown in Table 1.

**Figure 6:**
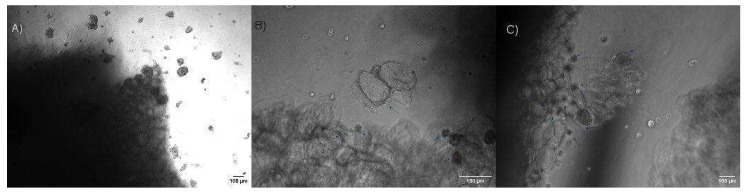
(**A**) Interactions of cells with SDS, (**B**) SDS-GTMAC, and (**C**) Construct scaffolds at day 2; arrows show the interactions of cells with the SDS-GTMAC and Construct scaffolds.

**Figure 7:**
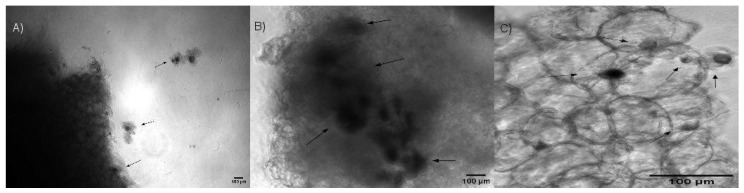
(**A**) Interactions of cells with SDS, (**B**) SDS-GTMAC, and (**C**) Construct scaffolds at day 8; arrows show the interactions of cells with the SDS-GTMAC and Construct scaffolds.

**Figure 8:**
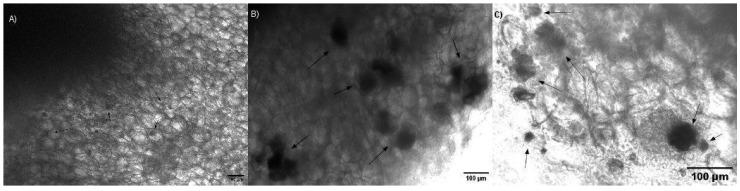
(**A**) Interactions of cells with SDS, (**B**) SDS-GTMAC, and (**C**) Construct scaffolds at day 15; arrows show the interactions of cells with the SDS-GTMAC and Construct scaffolds.

**Figure 9:**
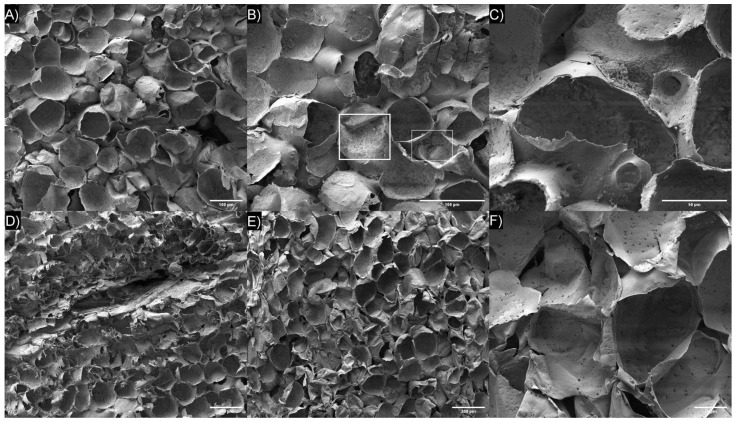
Interactions of hMSCs with SDS-treated scaffolds at day 7 of osteogenic differentiation. Cells in (**A**–**D**)**,** are indicated by black arrows. In (**B**), the white rectangles highlight regions of probable previous cell interactions with the scaffold, and the arrows indicate putative extracellular matrix deposition by hMSCs. (**D**) The xylem and phloem regions of the SDS-decellularized scaffold. (**E**,**F**) Negative-control SDS scaffolds without cell seeding.

**Figure 10:**
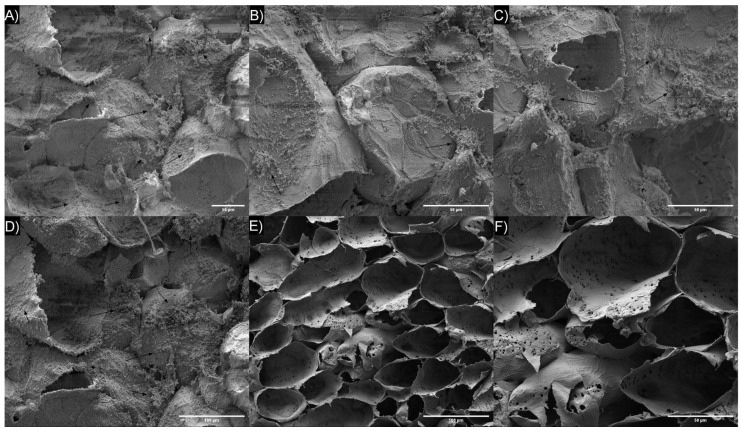
Interactions of cells with the SDS-GTMAC-treated scaffold at day 7 of hMSC differentiation. Cells in (**A**–**D**) are indicated by black arrows. (**E**,**F**) Negative-control SDS-GTMAC scaffolds without cell seeding.

**Figure 11:**
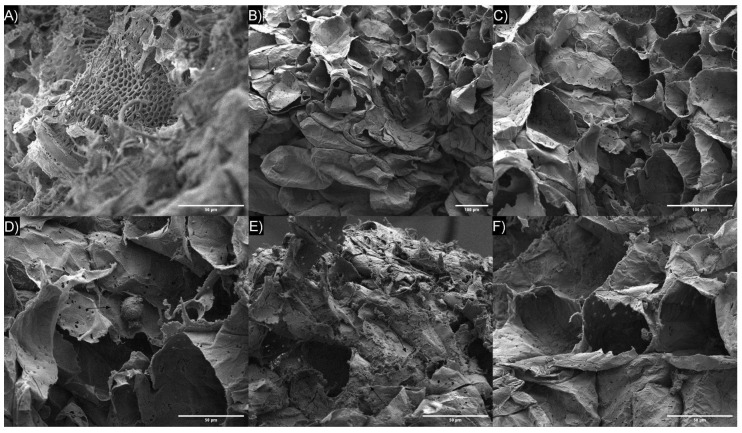
Interactions of cells with the Construct scaffold at day 7 of hMSC differentiation. Cell attachment in the scaffold was identified at different magnifications: Cells in (**B**–**E**) are indicated by black arrows. (**A**,**F**) Negative-control Construct scaffolds without cell seeding.

**Figure 12:**
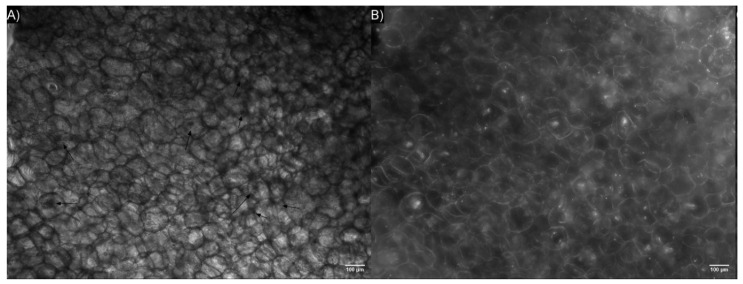
Fluorescence images of cell adhesion and interaction with the Construct scaffolds at day 7 of osteogenic differentiation: (**A**) brightfield image; (**B**) DAPI fluorescence.

**Figure 13:**
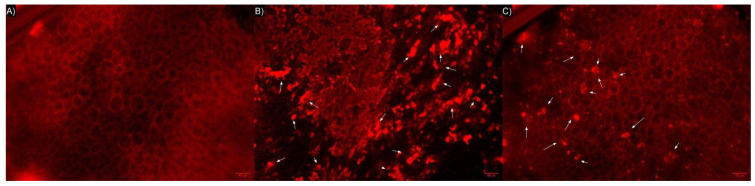
Fluorescence images of the GTMAC Alizarin-Red-stained scaffold at day 7 of osteogenic differentiation: (**A**) control scaffold without cells; (**B**) apparent calcified nodules in GTMAC-modified scaffold, shown with white arrows; (**C**) apparent calcified nodules in control osteogenic culture.

**Cell Culture in the Scaffold:** In Figure 6, Figure 7 and Figure 8, cell interactions with the SDS, SDS-GTMAC, and Construct groups were observed at days 2, 8, and 15 by brightfield light microscopy under osteoblast differentiation medium; dark regions in the SDS scaffold appear due to the thickness of the decellularized tissue and not the accumulation of cells. Supplementary Figure S2 shows the arrangement of osteoblasts in the SDS scaffold at day 2. In Supplementary Figure S3, a single cell was observed in the culture at day 15. SEM images in Figure 9, Figure 10 and Figure 11 reveal the morphology of the cells and their interactions with the SDS, SDS-GTMAC, and Construct scaffolds. Cells interacting with the SDS-GTMAC scaffold under DAPI fluorescence are shown in Figure 12, and Figure 13 shows Alizarin Red staining in the GTMAC-modified scaffold; brilliant bodies can be seen under fluorescence at 520 nm.

## 4. Discussion

As a novel biomaterial, the decellularized corn cob scaffold supports mesenchymal stem cell adhesion, likely mediated by electrostatic interactions arising from the cationized surface. The decellularization process is cost-effective and improves biocompatibility by removing potentially cytotoxic molecules from the native tissue. Although it reduced the mechanical properties of the scaffold—partly attributed to the partial loss of lignin, cellulose, and hemicellulose, which are the main structural components conferring mechanical integrity to the native tissue—it did not significantly alter its surface topology or compromise its cell adhesion capacity. Furthermore, the cellulosic nature of this plant-derived tissue makes it chemically tunable—in this study, functionalization with GTMAC was key to promoting cell adhesion.

When decellularizing the plant tissue, two critical challenges arise: insufficient microporosity to support cell adhesion, and cytotoxicity derived from phenolic compounds inherent to the native tissue, including salicylic acid [43]. Among the chemical agents evaluated, both SDS and NaOH proved effective at achieving decellularization; however, despite NaOH being a highly efficient option, the potential generation of residual phenolic byproducts represents a risk that may compromise the biocompatibility of the resulting scaffold [9,44]. In contrast, SDS presents a more favorable profile for plant tissue decellularization, as its residues can be effectively eliminated through a CaCl_2_ salt buffer treatment that induces phase separation of the detergent, allowing it to wash out of the scaffold [45]; this results in significantly higher cell viability compared to untreated SDS scaffolds [45]. While chemical decellularization agents broadly carry the risk of altering ECM biochemical composition and introducing cytotoxic residues [46], the removability of SDS residues positions it as the safer and more biocompatible choice for plant-derived scaffold preparation.

Plant-derived scaffolds have emerged as a promising alternative for 3D cell culture; however, their native cellulosic matrix inherently lacks cell adhesion motifs, limiting their direct use with adhesive cell types. To address this limitation, we exploited the chemical tunability of cellulose by alkalinizing the surface to expose hydroxyl groups, which were subsequently functionalized with GTMAC to introduce quaternary ammonium groups capable of promoting electrostatic cell adhesion. FTIR analysis confirmed the functionalization, showing increased absorbance in bands corresponding to cellulose, hemicellulose, and the quaternary ammonium groups; the elevated absorbance of cellulose and hemicellulose bands may reflect partial degradation of these components induced by NaOH treatment. Additionally, a color change was observed upon NaOH addition during functionalization, and the adherence of phenol red to the scaffold walls following GTMAC treatment suggests electrostatic interactions between the positively charged surface and anionic molecules, further supporting the success of the functionalization.

Beyond improving cell adhesion, these chemical modification strategies may also enhance the mechanical properties of the scaffold, broadening its potential applications. In particular, mechanically reinforced scaffolds capable of withstanding higher compressive loads could provide mechanoregulatory stimuli that favor osteogenic differentiation and calcium phosphate deposition, representing a promising avenue for bone tissue engineering.

MTT assays provide an approach to evaluate the viability of cells exposed to decellularized scaffolds; however, viability was reduced in cells exposed to GTMAC-modified scaffolds. The decreased absorbance values may be attributed to several factors: probable cell death caused by residues from the chemical functionalization reaction, the intrinsic membrane-disrupting properties of quaternary ammonium groups—which are known to act as surfactants—or cell redistribution onto the scaffold surface upon contact, reducing the number of cells remaining on the plate. To investigate the latter hypothesis, the GTMAC-modified scaffold was transferred to a new plate following the MTT assay; subsequent culture revealed apparent cell presence on the new plate surface (Supplementary Figure S4), consistent with scaffold-mediated cell redistribution rather than true cytotoxicity. Whether this redistribution involves active migration or passive transfer cannot be determined from the current data; a scratch healing assay is proposed as a necessary experiment in future work to address this question. A live–dead assay should be performed to confirm the presence of cells in the scaffold.

The relative elastic modulus of the corn cob scaffold confirms its elastic behavior under compression along its axial direction. The results demonstrate that both decellularized and autoclaved scaffolds exhibited decreased mechanical properties compared to the native tissue, which was expected given that high concentrations of SDS and autoclaving are known to disrupt plant cell walls and degrade structural components. Autoclaving exposes the primary structural components of the cell wall—cellulose, lignin, and hemicellulose—to elevated temperatures and pressures, compromising their mechanical integrity and altering the structural properties of the scaffold. This is consistent with the FTIR analysis, which revealed a reduction in absorbance at 1035 cm^−1^ and 1155 cm^−1^, corresponding to C–O–C stretching vibrations of cellulose and hemicellulose, respectively, confirming partial thermal degradation of these structural polysaccharides. Notably, the reduction in Young’s modulus not only reflects mechanical degradation but also suggests an increase in tissue elasticity, further supported by compression assays in which all three scaffold groups recovered over 80% of their original length upon load removal, demonstrating predominantly elastic mechanical behavior. Regarding sterilization, autoclaving was selected as the method of choice, as antibiotic–antifungal treatment proved insufficient, and ethanol-based sterilization risked irreversible dehydration of the scaffold matrix, compromising its capacity to absorb culture media. However, it must be acknowledged that the FTIR analysis revealed a partial reduction in quaternary ammonium group absorbance at the 1470–1490 cm^−1^ region following autoclaving of the GTMAC-functionalized scaffold, suggesting that the sterilization process may have partially compromised the chemical modifications introduced during functionalization. As cell seeding was performed on autoclaved scaffolds, the surface chemistry presented to the hMSCs during culture may not fully reflect the functionalization achieved prior to sterilization. Future work should, therefore, evaluate the extent to which residual GTMAC functionalization is sufficient to support cell adhesion or, alternatively, investigate sterilization methods that better preserve surface chemistry, such as UV irradiation. It should also be noted that this mechanical testing was conducted under hydrated conditions, with a scaffold water content of 232.90%, to simulate the physiological environment experienced by cells during in vitro culture. As reported for corn-cob-derived materials [10], hydration reduces compressive stiffness relative to the dry state; the modulus values obtained in the present study should, therefore, be interpreted as physiologically relevant estimates rather than intrinsic dry-state mechanical properties [47]. Paired dry/wet testing is proposed as a priority in future characterization work.

Mesenchymal stem cells were seeded onto the scaffolds following trypsinization from the culture flask. Cells were then transferred to SDS-decellularized, GTMAC-functionalized, and hydrogel-functionalized scaffolds. At day 2, 80% confluency was observed in the well plates; images were taken at day 4, corresponding to day 2 of osteoblast differentiation medium treatment. It is observed that, in all three scaffolds throughout the 15 days, cell adhesion was allowed, and at days 8 and 15, scaffolds showed a similar cell arrangement to that reported in Lee’s research [14]. Limited cell proliferation was observed across all three scaffold groups, as brightfield microscopy did not reveal a notable increase in cell-occupied areas over the observation period. However, it should be noted that proliferation was not quantitatively assessed in the present study. SEM images in Figure 9, Figure 10 and Figure 11 show all three scaffold types at day 7 of osteoblast differentiation. Preosteoblast-like cells are observed in the SDS and SDS-GTMAC scaffolds, indicated by black arrows. In Figure 9B, lentil-shaped structures are visible on the plant-derived scaffold surface, suggesting prior cell interaction or detachment. Cell presence in the Construct scaffold was limited under SEM imaging, with only a single cell identified across the analyzed sections. This may reflect suboptimal hydrogel coating adhesion, warranting further optimization of the coating strategy. Alternatively, and perhaps more likely, the SEM sample preparation process—including dehydration and sputter coating—may have resulted in cell detachment. A greater number of cells undergoing osteogenesis were observed in the SDS-GTMAC scaffold compared to the SDS scaffold, supported by visible extracellular matrix deposition on the scaffold surface. The SDS-GTMAC scaffold in Figure 12 shows DAPI-stained nuclei interacting at day 7 with vegetal cell walls and hMSCs/preosteoblasts, suggesting clear cell adhesion and maintenance on the vegetal scaffold. Discrete calcium nodules were observed under Alizarin Red S fluorescence microscopy in the GTMAC-functionalized scaffold, with excitation at 530–560 nm and emission at 580–620 nm. The calcium nodule signal was distinguishable from scaffold autofluorescence by its comparatively greater intensity. Mineralization appeared less extensive in the three-dimensional scaffold compared to the 2D osteogenic culture shown in Figure 13, which is consistent with the expected differences in nutrient diffusion and matrix maturation between two-dimensional and three-dimensional culture systems.

## 5. Conclusions

The present study demonstrates, for the first time, the use of decellularized *Zea mays* (corn cob) as a three-dimensional scaffold for cell culture, representing a novel and accessible plant-derived biomaterial for bone tissue engineering applications. GTMAC functionalization of the SDS-decellularized scaffold was shown to improve cell interactions, while further research must assess the effectiveness of the alginate–gelatin hydrogel coating to enhance cell adherence to the scaffold surface, collectively supporting mesenchymal stem cell viability throughout the culture period.

These findings suggest that the GTMAC scaffold—comprising the decellularized and GTMAC-functionalized scaffold—holds potential as a platform for osteoblastic cell culture and, prospectively, for bone tissue modeling. Nevertheless, several limitations must be acknowledged. The current study was conducted in vitro, and the long-term stability of the hydrogel coating, as well as the osteogenic differentiation capacity of seeded cells, warrants further investigation. Quantitative assessment of cell proliferation and matrix mineralization would strengthen the conclusions drawn from the microscopy-based observations presented here. The present study supports the cytocompatibility of the decellularized *Zea mays* scaffold and its capacity to support MSC adhesion under osteogenic culture conditions, but does not provide sufficient biochemical or transcriptional evidence to confirm osteogenic differentiation.

Future work should focus on quantifying cell adherence by incorporating DNA-based assays (e.g., PicoGreen) or direct cell counting of the supernatant at 24 h post-seeding to provide a rigorous seeding efficiency metric. Confirming osteogenic differentiation through established biochemical markers, such as an alkaline phosphatase (ALP) activity assay and antibody immunofluorescence, is recommended, as well as scaling the scaffold fabrication process, and evaluating the construct in more complex culture systems. In vivo validation will ultimately be necessary to assess the translational potential of this biomaterial. Taken together, these results establish *Zea mays*-derived scaffolds as a promising, low-cost, and sustainable alternative in the bone tissue engineering landscape.

## Figures and Tables

**Table 1:** Elastic moduli of control, sterilized control, and SDS groups obtained through axial quasi-static loading.

Axial Quasi-Static Loading			
Groups	Control	Sterilized Control	SDS
E (kPa)	546.56	254.00	336.71

## Data Availability

The original contributions presented in this study are included in the article/Supplementary Materials. Further inquiries can be directed to the corresponding author.

## Supplementary Materials

The following supporting information can be downloaded at: https://www.mdpi.com/article/10.3390/jfb17060267/s1. Table S1: Primers used to evaluate osteogenesis after 15 days. Figure S1. Representation of the initial behaviour of the corn cob under axial compression in control (A), control autoclaved (B), and SDS decellularized (C) corn cobs. Figure S2. SDS decellularized pith scaffold hosting DAPI stained mesenchymal stem cells adhered to the surface. Figure S3. Construct scaffold hosting mesenchymal stem cells exposed to osteoblast media adhered to the surface at day 15. Figure S4. Cells getting out of the SDS-GTMAC scaffold at day 2 of translation into a new plate.
