# Supplementary material for: Development of a Scaffold from the Cob of Zea mays L. “Choclo” to Obtain an In Vitro Bone Tissue Model
Source: J Funct Biomater. 2026 Jun 1;17(6):267. doi: 10.3390/jfb17060267 (PMC13301072; doi:10.3390/jfb17060267)
Supplement: Supplementary file 1 [file jfb-17-00267-s001.zip › jfb-4246451-supplementary.pdf]

Supplementary Material:

SUPPLEMENTARY TABLE:

	FORWARD	REVERSE
Col-1	CAGCCGCTTCACCTACAGC	TTTTGTATTCAATCACTGTCTTGCC
Beta-ACTIN	TCCCTCGGAGAAGAGCTACG	GTAGTTTCGTGGATGCCACA
GAPDH	TTGGCTACAGCAACAGGGTG	GGGGAGATTCAGTGTGGTGG
ALP	GCTGTAAGGACATCGCCTACCA	CCTGGCTTTCTCGTCACTCTCA
RunX2	AACCCACGAATGCACTATCCA	CGGACATACCGAGGGACATG
Osteacalcina	CTCACACTCCTCGCCCTATTG	GCCTGGGTCTCTTCACTACCT

Table S1. Primers used to evaluate osteogenesis after 15 days.

SUPPLEMENTARY FIGURES:

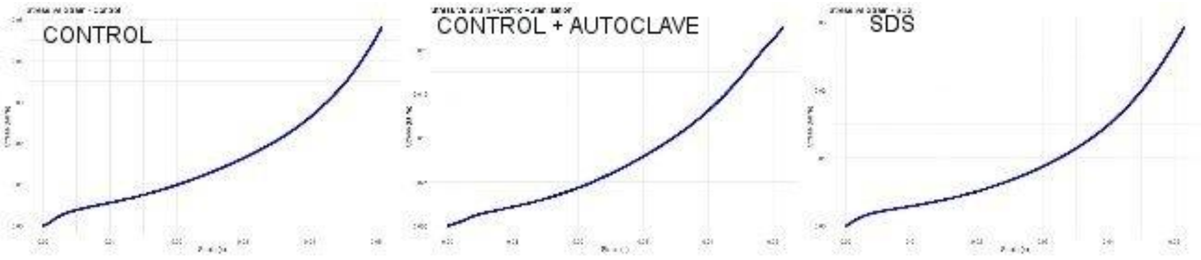

Figure S1. Representation of the initial behaviour of the corn cob under axial compression in control (A), control autoclaved (B), and SDS decellularized (C) corn cobs.

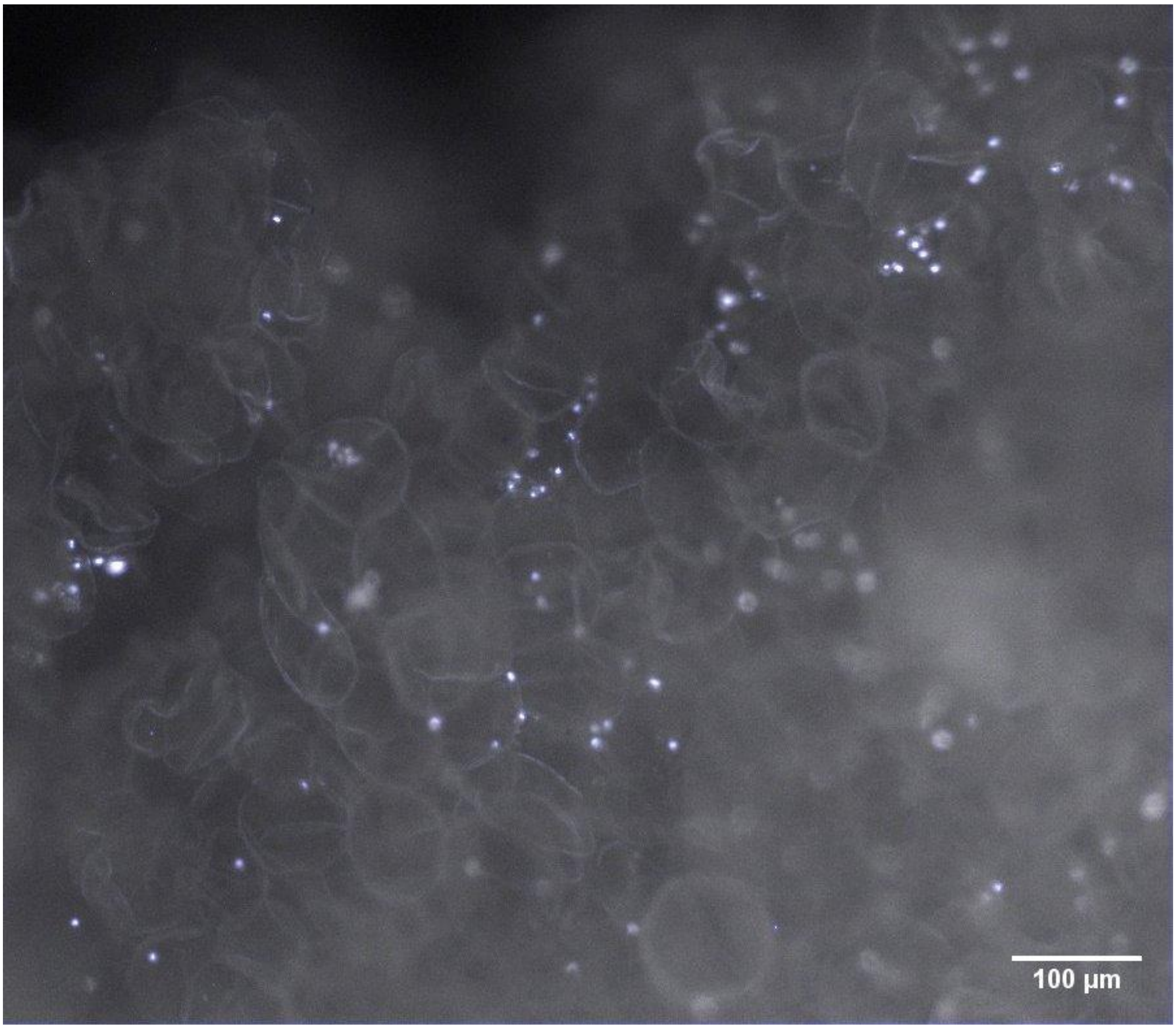

Figure S2. SDS decellularized pith scaffold hosting DAPI stained mesenchymal stem cells adhered to the surface.

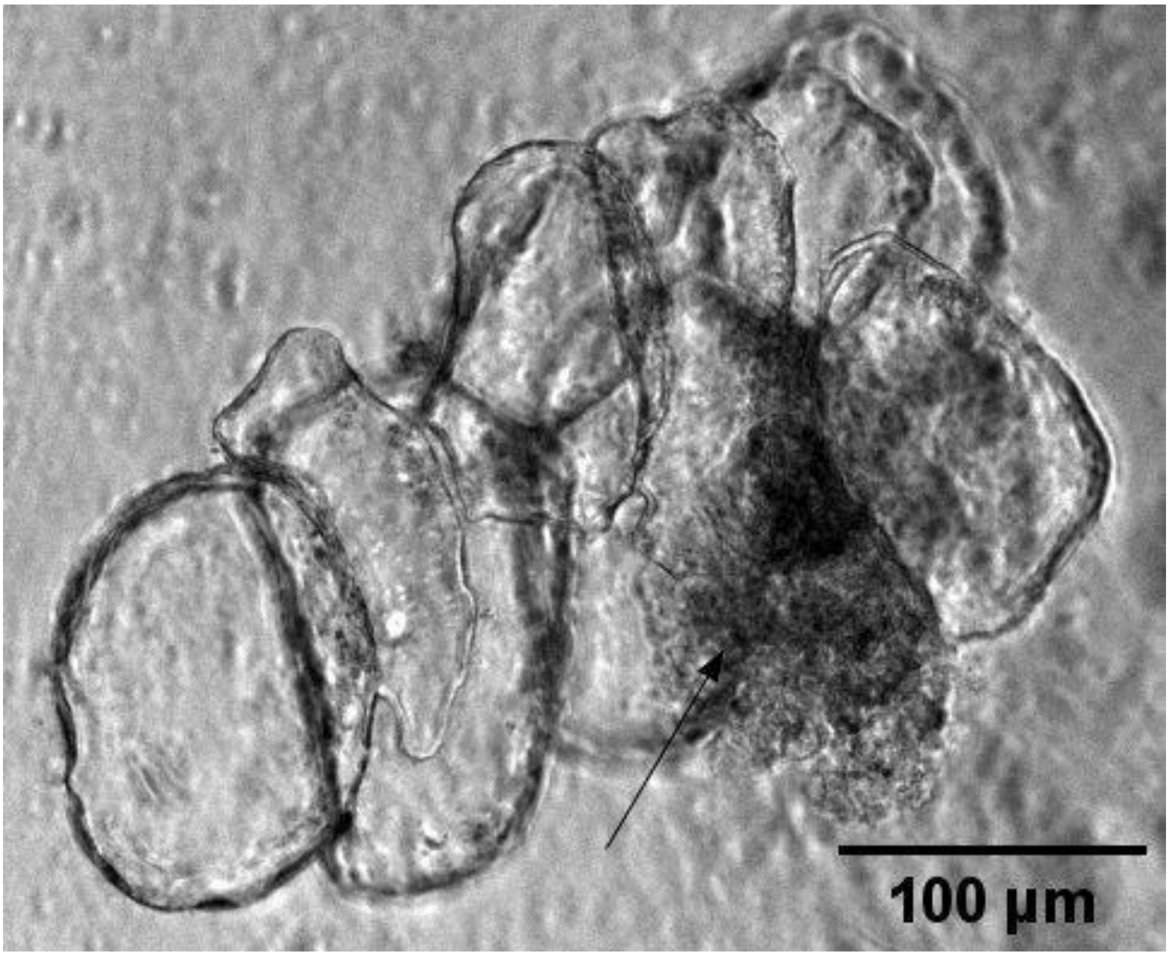

Figure S3. Construct scaffold hosting mesenchymal stem cells exposed to osteoblast media adhered to the surface at day 15.

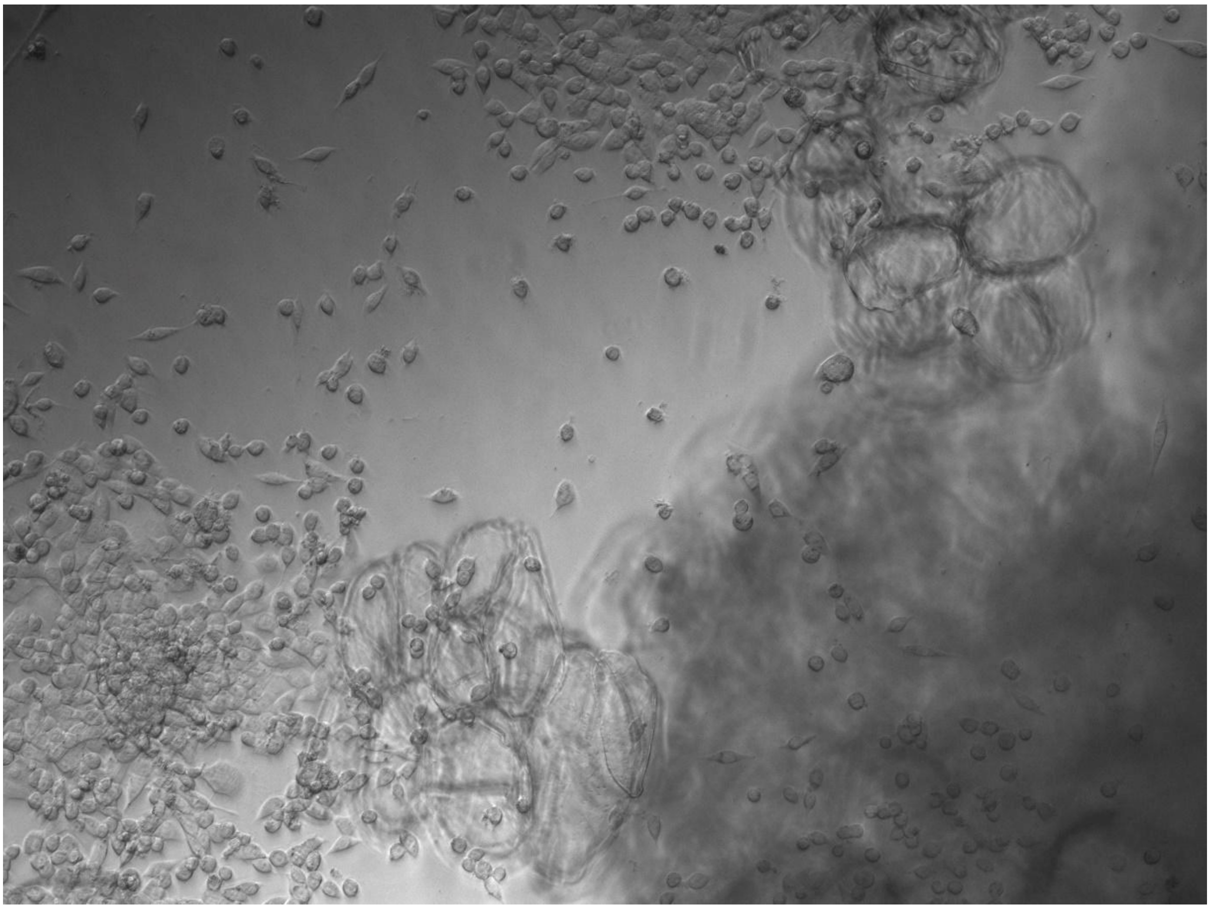

Figure S4. Cells getting out of the SDS-GTMAC scaffold at day 2 of translation into a new plate.
